# TREM-1 promotes intestinal tumorigenesis

**DOI:** 10.1038/s41598-017-14516-4

**Published:** 2017-11-01

**Authors:** Leslie Saurer, Daniel Zysset, Silvia Rihs, Lukas Mager, Matteo Gusberti, Cedric Simillion, Alessandro Lugli, Inti Zlobec, Philippe Krebs, Christoph Mueller

**Affiliations:** 1 Institute of Pathology, University of Bern, Bern, Switzerland; 2 Department of Physiology and Pharmacology, Cumming School of Medicine, University of Calgary, Calgary, Canada; 3 Department of BioMedical Research, University of Bern, Bern, Switzerland; 4 Interfaculty Bioinformatics Unit and SIB Swiss Institute of Bioinformatics, University of Bern, Bern, Switzerland

## Abstract

Triggering receptor expressed on myeloid cells-1 (TREM-1) is a potent amplifier of pro-inflammatory innate immune responses. Increasing evidence suggests a role for TREM-1 not only in acute pathogen-induced reactions but also in chronic and non-infectious inflammatory disorders, including various types of cancer. Here, we demonstrate that genetic deficiency in *Trem1* protects from colorectal cancer. In particular, *Trem1* ^−/−^ mice exhibited reduced tumor numbers and load in an experimental model of inflammation-driven tumorigenesis. Gene expression analysis of *Trem1* ^−/−^ versus *Trem1* ^+/+^ tumor tissue demonstrated distinct immune signatures. Whereas *Trem1* ^−/−^ tumors showed an increased abundance of transcripts linked to adaptive immunity, *Trem1* ^+/+^ tumors were characterized by overexpression of innate pro-inflammatory genes associated with tumorigenesis. Compared to adjacent tumor-free colonic mucosa, expression of *Trem1* was increased in murine and human colorectal tumors. Unexpectedly, TREM-1 was not detected on tumor-associated Ly6C^−^ MHC class II^+^ macrophages. In contrast, TREM-1 was highly expressed by tumor-infiltrating neutrophils which represented the predominant myeloid population in *Trem1* ^+/+^ but not in *Trem1* ^−/−^ tumors. Collectively, our findings demonstrate a clear role of TREM-1 for intestinal tumorigenesis and indicate TREM-1-expressing neutrophils as critical players in colorectal tumor development.

## Introduction

Inflammation is now considered one of the hallmarks of cancer^1^. While a tumor-elicited inflammatory response can be observed for most malignancies, colorectal cancer (CRC) represents a paradigm for cancer that is substantially promoted by chronic inflammation^2^. For instance, patients with ulcerative colitis have an increased propensity to develop colorectal carcinomas and the long-term use of non-steroidal anti-inflammatory drugs effectively reduces colon cancer risk^2^. In mice, CRC can be modeled by the induction of microbial-driven chronic intestinal inflammation^3,4^. However, also in genetic models of CRC, early tumor-elicited intestinal inflammation represents a major driver of tumor growth^5^.

Despite the relation between intestinal inflammation and CRC, increased expression of CD8 T cell-, T follicular helper (Tfh) cell- and B cell-associated markers in human CRC is linked to a favorable clinical prognosis, highlighting the importance of adaptive anti-tumor immunity^6^. On the other hand, cells and mediators of the innate immune system take a critical role in promoting tumor development and progression. Innate cytokines such as TNF, IL1β, IL-6, IL-22 can directly act on intestinal epithelial cells to activate NF-κB-dependent oncogenic pathways, to promote WNT-β-catenin or STAT3-dependent proliferation and to induce mesenchymal and stemness features^7^. Inhibition of these cytokines, their downstream molecules or depletion of their cellular sources has been shown to prevent and ameliorate tumor development in various murine models of CRC^8–13^. While pro-inflammatory cytokines thus represent attractive targets for immune-based therapies, a major impediment are their relatively redundant effects in the activation of downstream signaling pathways. Moreover, innate cytokines not only exert disease-promoting but also tissue-protective functions. The latter aspect seems to be of particular importance in the gastrointestinal tract where pro-inflammatory mediators and pathways critically contribute to effective epithelial repair and microbial control. Thus, any treatment strategies aiming at cancer-related inflammation in CRC face the challenge of identifying pathways that can be specifically targeted without compromising tissue homeostasis or generally jeopardizing anti-microbial and anti-tumor immunity^2^.

Our laboratory has a longstanding interest in TREM-1, an activating member of the Ig superfamily that is exclusively expressed on neutrophils and subsets of monocytes and macrophages^14^. Activation of TREM-1 through its yet incompletely defined ligands, so far comprising HMGB1^15,16^ and multimerized peptidoglycan recognition protein-1 (PGLYRP)^17^, potently amplifies oxidative burst and pro-inflammatory cytokine production, particularly when signaling in concert with other pattern recognition receptors^18^. Whereas the role of TREM-1 has initially been acknowledged from models of septic shock where blockade of TREM-1 conferred significant protection^19^, increasing evidence suggests an involvement of TREM-1 also in chronic and non-infectious inflammatory conditions^20–23^. We have previously demonstrated that TREM-1-expressing cells accumulate in the inflamed intestinal mucosa of patients with inflammatory bowel diseases (IBD) and that TREM-1-mediated signaling critically contributes to intestinal inflammation^24^. Accordingly, *Trem1*-deficient (*Trem1* ^−/−^) mice exhibited significantly reduced clinical and histopathological signs of experimental colitis^25^. Strikingly, and in contrast to *Tnf*-deficient mice^26^, *Trem1* ^−/−^ mice were not impaired in their capacity for microbial control during dextran-sodium sulfate (DSS)-induced colitis or during distinct models of infection^25^. The restricted expression of TREM-1 on myeloid cells and its function as an amplifier rather than an initiator of inflammatory reactions indicate TREM-1 as an attractive upstream target for dampening disproportionate innate immune responses during chronic inflammation and malignant disease. Indeed, in human non-small cell lung cancer (NSCLC) increased TREM-1^+^ tumor-associated macrophages (TAMs) were linked with reduced survival, and inhibiting TREM-1 suppressed tumor growth in a mouse xenograft NSCLC model^27,28^. Likewise, TREM-1 was significantly involved in Kupffer cell activation and tumor development in a chemically-induced model of hepatocellular carcinoma (HCC)^16^.

In the present study, we hypothesized that TREM-1 contributes to colitis-associated cancer in the azoxymethane (AOM)/DSS model of CRC. In line with the findings of Zhou *et al*.^29^, we demonstrate that absence of TREM-1-mediated signaling in AOM/DSS-treated *Trem1* ^−/−^ mice significantly attenuates intestinal tumor development. In wildtype *Trem1* ^+/+^ mice, *Trem1* was expressed in tumors but not in the tumor-free adjacent mucosa. Moreover, while the immune signature of *Trem1* ^−/−^ tumors was skewed towards increased expression of B cell and Tfh-associated genes, *Trem1* ^+/+^ tumors exhibited an increased abundance of neutrophils and overexpression of several innate pro-inflammatory genes that are typically associated with colorectal tumorigenesis. Our study further reveals that in contrast to the reported expression of TREM-1 on macrophages in NSCLC and HCC^16,27^, in murine CRC tumor-infiltrating neutrophils but not macrophages express TREM-1. Collectively, our findings indicate a disease-promoting role for TREM-1-expressing neutrophils in colitis-associated cancer.

## Results

### Deficiency in *Trem1* attenuates colitis-associated tumorigenesis

**Figure 1:**
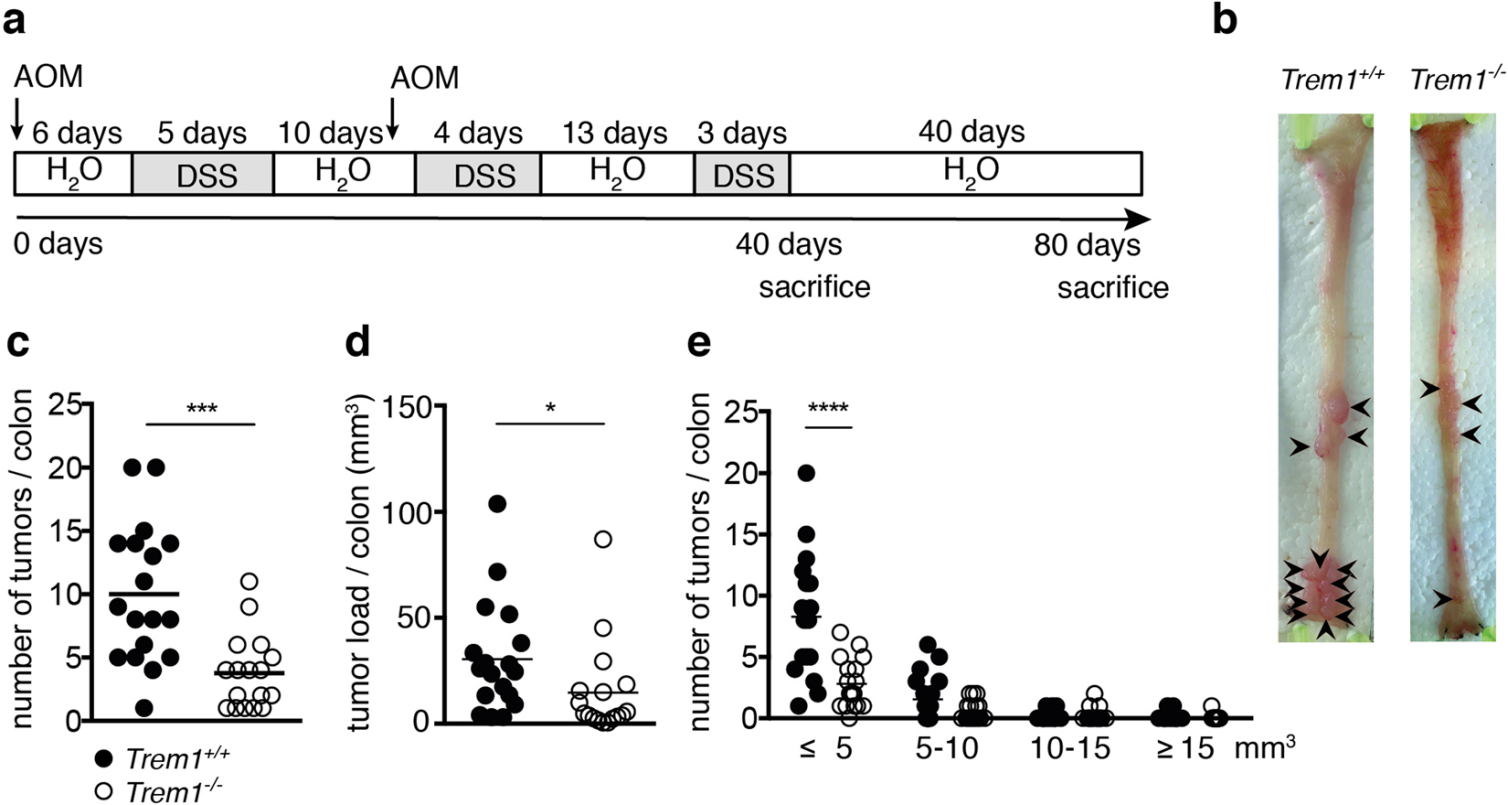
Deficiency in TREM-1 attenuates colitis-associated tumorigenesis. Mice were subjected to AOM/DSS and analyzed 80 days post initial AOM injection **(a)** Scheme of treatment protocol. **(b)** Macroscopic pictures of representative colons of *Trem1* ^+/+^ and *Trem1* ^−/−^ mice. Arrow heads indicate macroscopically visible tumors. **(c,d)** Number of tumors and total tumor load per colon. **(e)** Tumor size distribution: Number of tumors within the indicated size category per colon. **(c,d)** Symbols represent data for individual *Trem1* ^+/+^ (n = 18) and *Trem1* ^−/−^ (n = 17) mice, lines indicate mean values per group. Pooled data from two independent experiments are shown. Statistical testing was performed using the unpaired t-test **(c)**, the Mann Whitney t test **(d)** and 2way ANOVA **(e)**. ***p < 0.001; ****p < 0.0001.

To address the role of TREM-1 in CRC development, we used *Trem1*-deficient mice (*Trem1* ^−/−^) mice that were generated in our laboratory^25^ and the AOM/DSS model of murine CRC^3^. *Trem1* ^−/−^ and *Trem1* ^+/+^ mice were injected twice with the procarcinogen AOM and subjected to three cycles of DSS administration, followed by an additional latency period to allow for visible tumor growth (Fig. 1a). As anticipated from our previous studies^25^, *Trem1* ^−/−^ mice exhibited reduced body weight loss after DSS treatment and decreased colon shortening at the final time-point compared to *Trem1* ^+/+^ mice (Supplementary Figure 1a,b). Tumors were observed in both groups of mice (Fig. 1b). However, *Trem1* ^−/−^ mice displayed significantly fewer tumors with an average of 4 tumors per colon as opposed to 10 tumors per colon in the *Trem1* ^+/+^ group (Fig. 1c). In line with this observation, the overall tumor load in *Trem1* ^−/−^ mice was significantly lower compared to *Trem1* ^+/+^ mice (Fig. 1d). Analysis of tumor size distribution indicated that presence of TREM-1 mostly impacted on the incidence of small tumors (≤5 mm^3^) rather than affecting the growth of large tumors (Fig. 1e). Taken together, TREM-1 significantly contributed to colitis-associated tumor development.

### Distinct immune transcriptome in *Trem1*^+/+^ versus *Trem1*^−/−^ tumors

**Figure 2:**
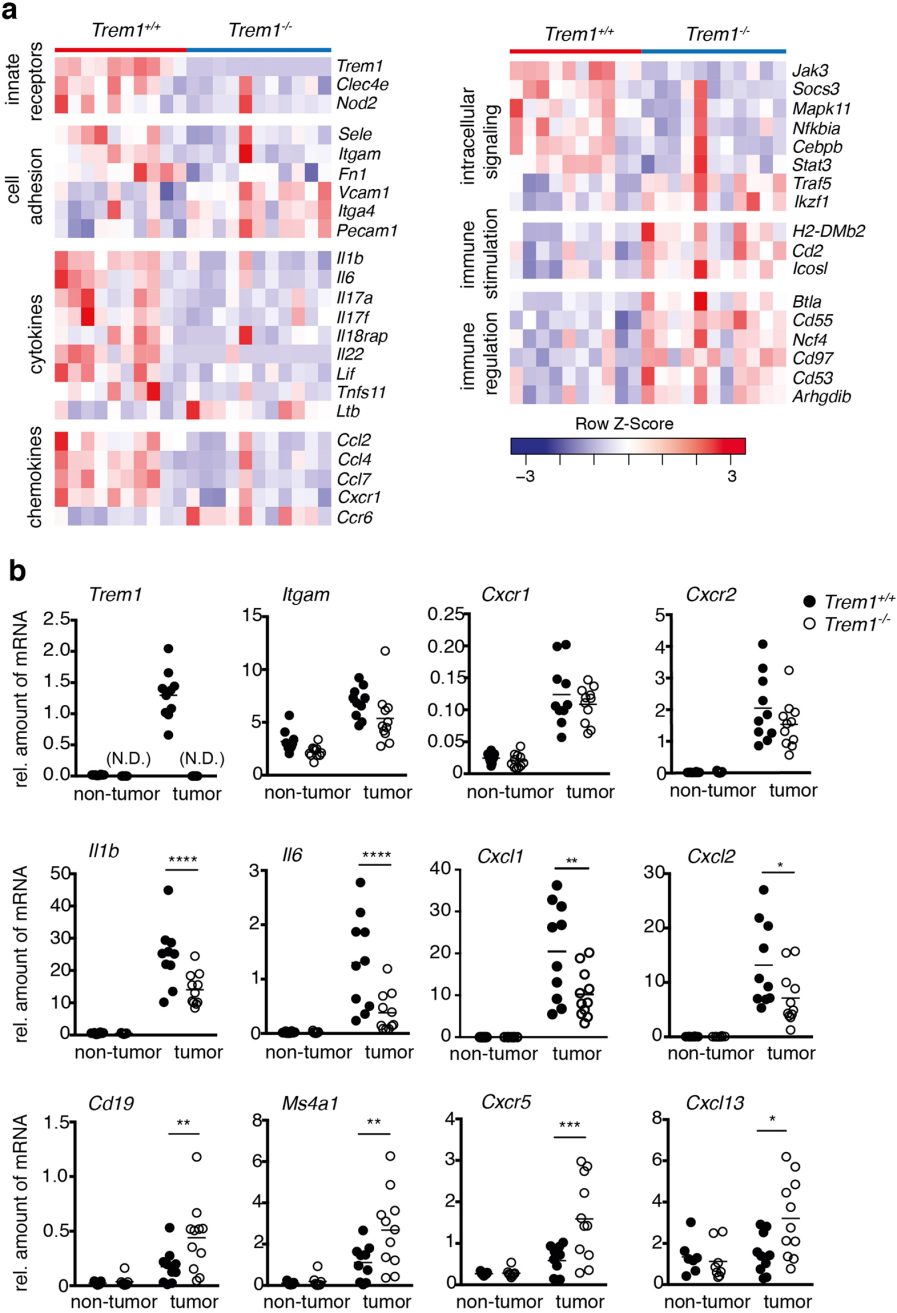
Distinct immune transcriptome in *Trem1* ^+/+^ versus *Trem1* ^−/−^ tumors. **(a)** Heat-map of functionally defined subsets of mRNA transcripts that are differentially expressed in *Trem1* ^+/+^ tumors (n = 10) compared to *Trem1* ^−/−^ (n = 11) tumors as determined by NanoString-based gene expression profiling. **(b)** qRT-PCR-based analysis of functionally defined gene groups in tumors versus adjacent tumor-free colonic tissues from *Trem1* ^+/+^ (n = 10) versus *Trem1* ^−/−^ mice (n = 11). Tumors were collected from individual mice at 80 days post initial AOM injection; from each mouse one tumor and one piece of tumor-free colonic mucosa were analyzed. Symbols show relative gene expression values (normalized to *Gapdh*) for individual mice, lines indicate mean values per group. (N.D.), not determined. Statistical testing was performed using 2way ANOVA. *p < 0.05; **p < 0.01; ***p < 0.001; ****p < 0.0001.

In order to investigate the underlying mechanisms behind TREM-1-driven tumor development, H&E stained sections of *Trem1* ^+/+^ and *Trem1* ^−/−^ tumors were at first assessed for the tumor grade (Supplementary Figure 1c,d). While AOM-induced tumors typically follow the adenoma-carcinoma sequence described for human CRC, in AOM/DSS-treated C57BL/6 mice carcinomas are rarely observed^3^. In *Trem1* ^+/+^ and also *Trem1* ^−/−^ mice, most tumors represented advanced adenomas (Supplementary Figure 1c,d). Immunohistochemistry stainings for Ki-67 or pSTAT3 did not indicate apparent differences between the two groups of mice (data not shown). We next subjected tumors of comparable size distribution (Supplementary Figure 2a) to a comprehensive NanoString-based gene expression profiling. Strikingly, clear differences in the immune transcriptome of *Trem1* ^+/+^ versus *Trem1* ^−/−^ tumors were detected with 50 genes that were distinctly expressed (Fig. 2a and Supplementary Table 1). *Trem1* ^+/+^ tumors exhibited an increased abundance of transcripts associated with innate immune responses; in particular, mRNA expression of pro-inflammatory mediators linked to intestinal tumorigenesis such as *Il1b* ^13,30,31^, *Il6* ^9,10^, *Il17* ^32–35^, *Il22* ^11,12^, *Ccl2* ^36^ and *Cxcl2* ^37–39^ was augmented compared to *Trem1* ^−/−^ tumors (Fig. 2a,b). Pathway analysis further identified neutrophil chemotaxis, TNF signaling and cellular extravasation as major differentially regulated pathways (Supplementary Table 2). In contrast, *Trem1* ^−/−^ tumors exhibited increased mRNA expression of molecules associated with immune regulation or adaptive immune stimulation (Fig. 2a and Supplementary Table 1). Since the anti-tumoral immune activity to AOM/DSS-induced CRC tumors is mainly mediated by cytotoxic CD8^+^ T cells, whose function is controlled by regulatory FOXP3^+^ T cells (Tregs)^40^, we examined whether TREM-1 signaling negatively regulates the generation of Tregs in CRC lesions of *Trem1* ^+/+^ mice. However, absence of TREM-1 did not affect the expression of *Foxp3* transcripts in AOM/DSS CRC tumors (Supplementary Figure 2) thus, suggesting that Tregs did not contribute to the observed differences between these tumors (Supplementary Figure 2b). When comparing additional genes that were included in the NanoString mouse immunology panel, the expression of markers associated with neutrophils but not monocytes/macrophages was increased in *Trem1* ^+/+^ tumors whereas *Trem1* ^−/−^ tumors displayed augmented expression of markers associated with B cells or follicular helper T cells (Supplementary Figure 2). Importantly, the above-described differences in gene expression were only observed for *bona fide* intestinal tumors but not for adjacent tumor-free colonic mucosa (Fig. 2b). As seen for inflammatory genes such as *Il1b* and *Il6*, also the expression of *Trem1* was highly increased in *Trem1* ^+/+^ tumors as opposed to matched tumor-free colonic mucosa (Fig. 2b).

In summary, presence or absence of TREM-1 had a distinct impact on the intratumoral immune transcriptome. While *Trem1* ^+/+^ tumors exhibited a transcriptional pattern typically associated with the inflammatory microenvironment of colorectal tumors, the immune signature of *Trem1* ^−/−^ mice was reminiscent of the Tfh/B cell axis that has previously been associated with a favorable prognosis in human CRC^6^.

### Flow cytometry analysis indicates increased abundance of neutrophils in *Trem1*^+/+^ tumors

**Figure 3:**
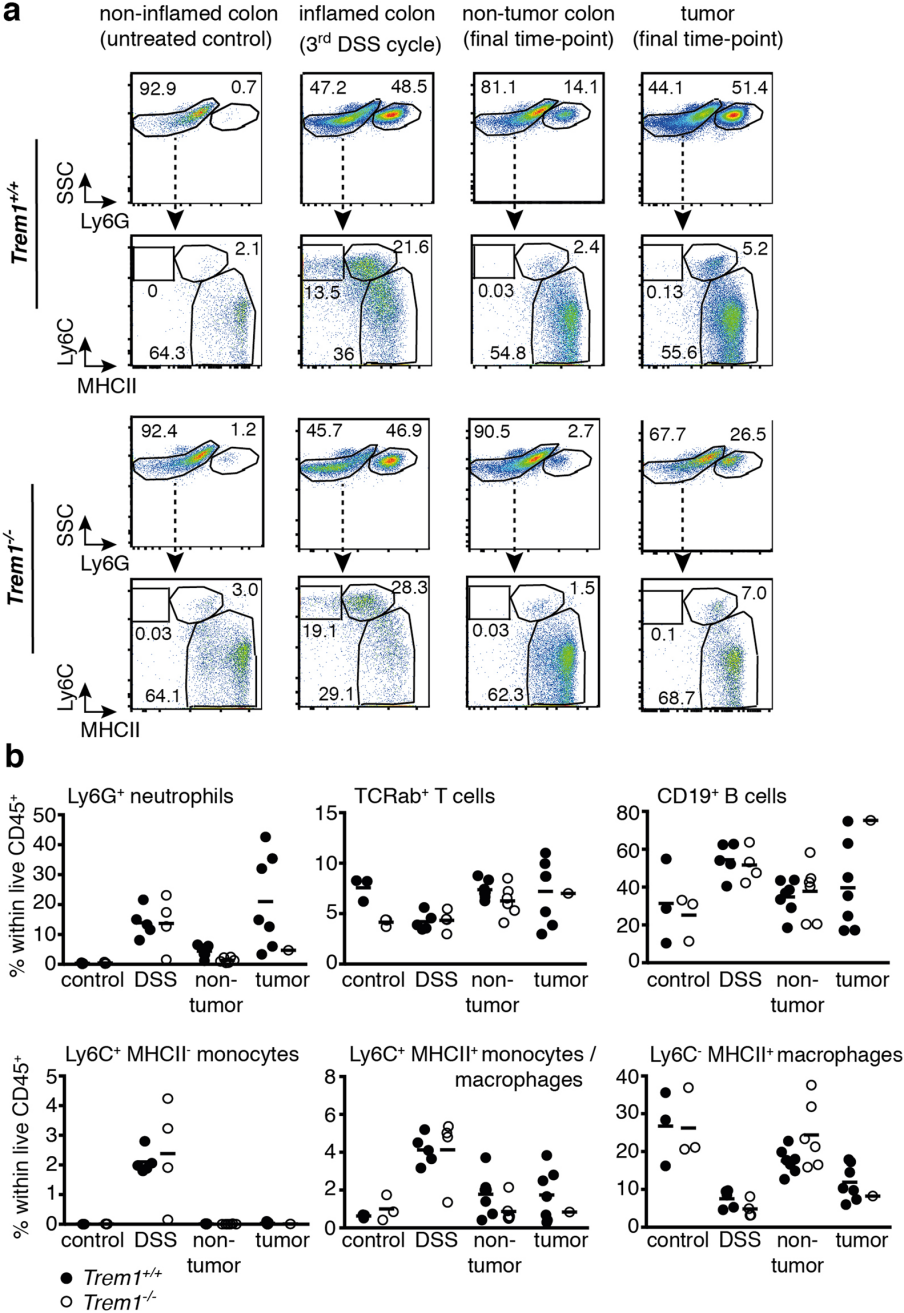
Increased abundance of tumor-infiltrating neutrophils in *Trem1* ^+/+^ mice. Colons from *Trem1* ^+/+^ and *Trem1* ^−/−^ mice were analyzed by flow-cytometry either after the 3^rd^ DSS cycle or at the final time-point. **(a)** Representative dot blots showing the relative frequency of live Ly6G^+^ neutrophils in CD11b^+^ cells and the “monocyte-waterfall”^64^ in live Ly6G^−^ CD11b^+^ cells. **(b)** Relative frequencies of the indicated cell subsets among live CD45^+^ cells isolated from tumor-free colonic mucosa or tumors. **(a,b)** Analysis at the final time-point included n = 3 age-matched untreated control mice per group. For AOM/DSS treated mice at the final time point, tumor-free colonic mucosa and tumors were analyzed separately. In the *Trem1* ^+/+^ group, tumors were pooled to yield one tumor sample per mouse; in the *Trem1* ^−/−^ group, tumors were pooled from n = 4 mice to yield a single tumor sample. Statistical testing was performed using 2way ANOVA.

We next aimed to assess the cellular infiltrates in *Trem1* ^+/+^ versus *Trem1* ^−/−^ tumors. *In situ* analysis of MPO (neutrophils), F4/80 (macrophages) and B220 (B cells) expression on immunostained tumor sections did not indicate apparent differences between the two groups of mice (data not shown). Therefore, we decided to use flow cytometry for a detailed characterization of the cellular infiltrates in *Trem1* ^+/+^ versus *Trem1* ^−/−^ tumors. We additionally included analysis of non-inflamed colons from untreated control mice, inflamed colons that were taken after the 3^rd^ DSS cycle, as well as tumor-free colonic mucosa at the final time-point. As anticipated, for non-inflamed colons no substantial differences between the two groups were observed; neutrophils and inflammatory Ly6C^+^ monocytes were hardly detectable and myeloid cells mostly consisted of resident Ly6C^-^ MHCII^+^ macrophages (Fig. 3a). After the 3^rd^ DSS cycle, colons from *Trem1* ^+/+^ and *Trem1* ^−/−^ mice showed a prominent increase in infiltrating neutrophils, Ly6C^+^ MHCII^−^ monocytes and also a subset of Ly6C^+^ MHCII^+^ cell representing monocyte/macrophage intermediates (Fig. 3a,b and see Supplementary Figure 3a for full gating strategy). At the end of the observation period, myeloid cell populations in the tumor-free colon were comparable to those seen in untreated non-inflamed controls; however, neutrophils were still present at elevated frequencies, particularly in *Trem1* ^+/+^ mice (Fig. 3a,b). Strikingly, in *Trem1* ^+/+^ tumors the relative abundance of neutrophils was even further increased, with Ly6G^+^ neutrophils representing up to 40% of tumor-infiltrating CD45^+^ leukocytes (Fig. 3a,b). While analysis of *Trem1* ^−/−^ tumors had to be restricted to a single sample of pooled tumors from several mice, the frequency of Ly6G^+^ neutrophils was clearly much lower (Fig. 3a,b). In contrast, *Trem1* ^−/−^ tumors exhibited a drastically higher proportion of CD19^+^ B cells (Fig. 3b). The differences in cellular infiltration were not related to tumor size, as the size distribution of tumors selected for flow cytometry was comparable between the two groups of mice (Supplementary Figure 4a,b). Moreover, there was no significant correlation between tumor size and abundance of neutrophil-associated transcripts (*Cxcr1*, *Cxcr2*) (Supplementary Figure 4c,d). Taken together, the flow cytometry analysis corroborated the notion that tumors of *Trem1* ^+/+^ and *Trem1* ^−/−^ mice mostly differed in the extent of tumor-infiltrating neutrophils versus B cells.

### Tumor-infiltrating neutrophils represent the predominant TREM-1-expressing myeloid cell subset

**Figure 4:**
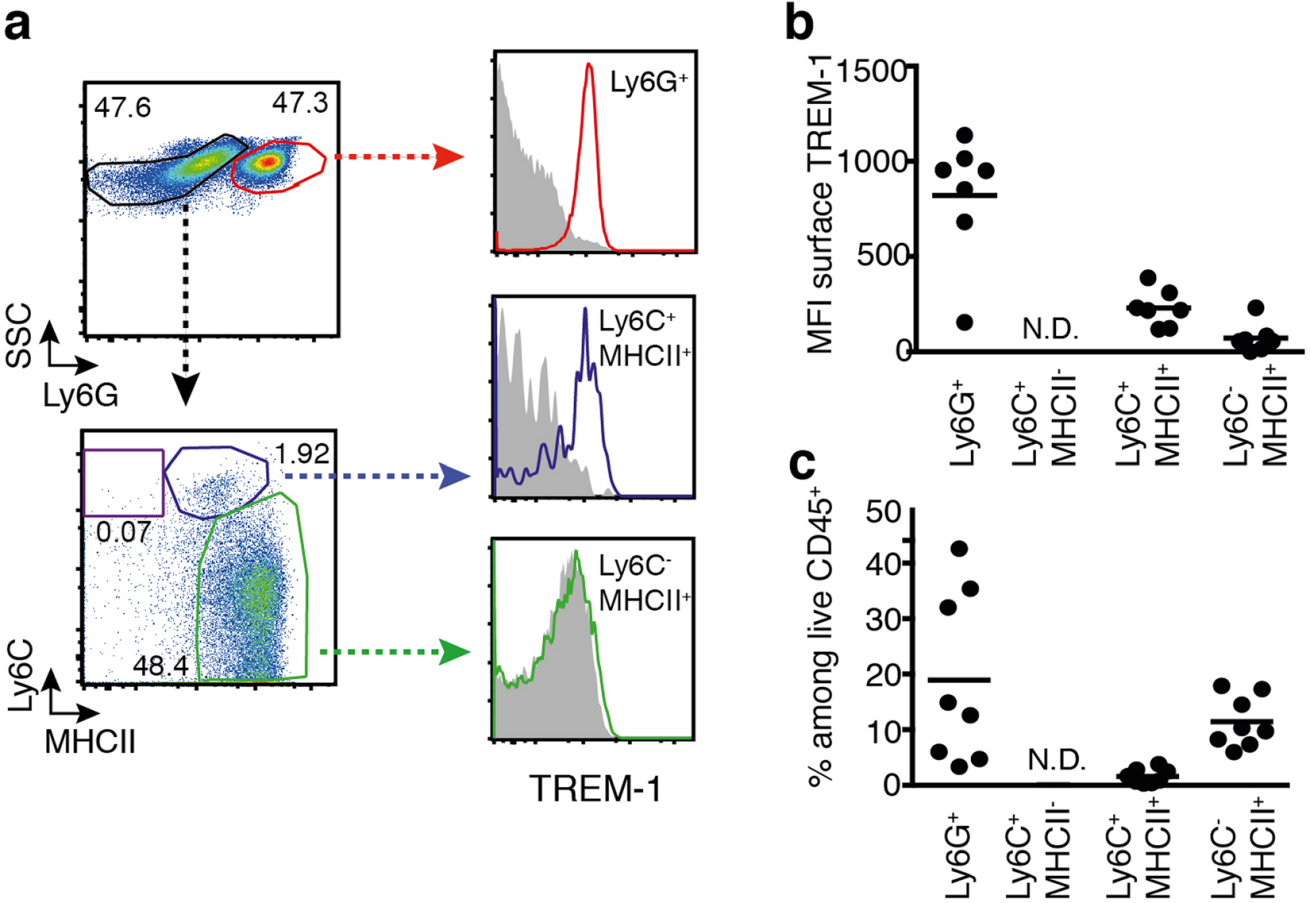
Tumor-infiltrating neutrophils but not tumor-associated macrophages express TREM-1. **(a)** Representative staining panels of a *Trem1* ^+/+^ tumor sample showing the relative frequency of live Ly6G^+^ neutrophils among tumor-infiltrating CD11b^+^ cells and the “monocyte-waterfall”^64^ in live Ly6G^−^ CD11b^+^ cells. Histograms show TREM-1 surface expression (lines; filled histograms represent isotype control-stained cells) in the indicated cell subsets. **(b)** Mean fluorescence intensity (MFI) of TREM-1 expression in the indicated cell subsets. **(c)** Relative frequency of the indicated cell subsets among live CD45^+^ tumor-infiltrating cells. Symbols represent data for individual mice (n = 7); lines show mean values per group. N.D., not detected.

Previous studies on the involvement of TREM-1 in hepatocellular carcinoma and lung cancer have suggested a role for TREM-1-expressing macrophages^16,27,41^. In the intestinal mucosa, TREM-1 is not detected on macrophages under homeostatic conditions^42^. While being highly expressed on infiltrating Ly6C^+^ monocytes and their Ly6C^+^ MHCII^+^ descendants in colitis, TREM-1 remains low on mature Ly6C^-^ MHCII^+^ macrophages even during inflammation (Supplementary Figure 3a,b)^43,44^. Since expression of TREM-1 can be induced by tumor-derived factors^41,45^, we nonetheless considered the possibility that macrophages in colorectal tumors expressed TREM-1. However, Ly6C^−^ MHCII^+^ macrophages in *Trem1* ^+/+^ tumors did not exhibit appreciable expression of TREM-1 (Fig. 4a,b). Whereas TREM-1 was present on Ly6C^+^ MHCII^+^ intermediate monocyte/macrophages, the relative abundance of these cells among CD45^+^ cells was very low (Fig. 4a,c). In contrast, TREM-1 was highly expressed on tumor-infiltrating Ly6G^+^ neutrophils which also represented the predominant myeloid cell subset in *Trem1* ^+/+^ tumors (Fig. 4a–c).

### TREM1 is expressed in human colorectal cancer

**Figure 5:**
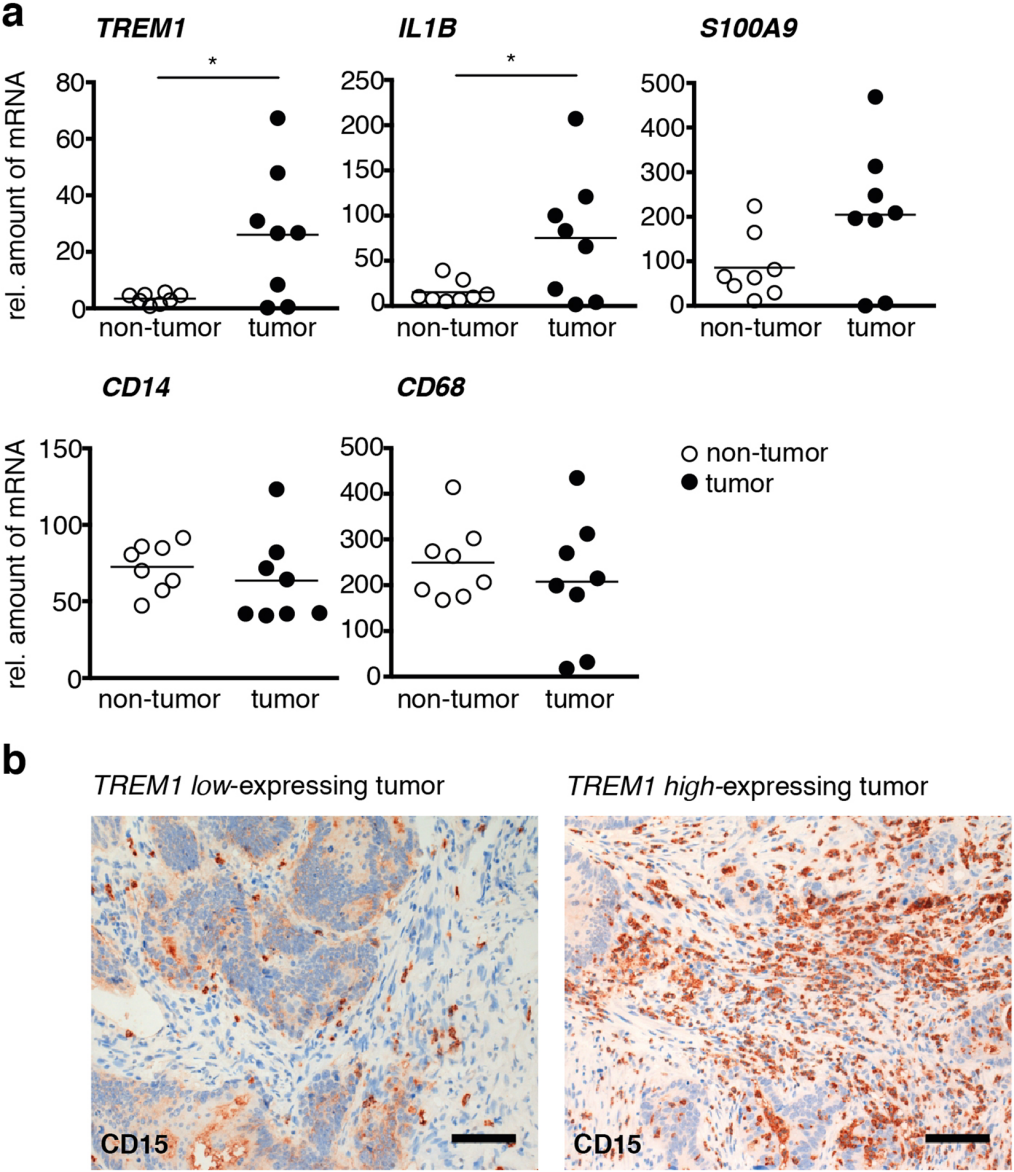
*TREM1* is expressed in human colorectal tumors. **(a)** Expression of *TREM1* and *IL1B* as well as *S100A9*, *CD14* and *CD68* was determined in human colorectal cancer tissue and matched tumor-free mucosa by qRT-PCR. Expression of genes of interest was normalized to the expression of *GAPDH*. **(b)** Representative images of tumor sections stained for the granulocyte marker CD15. One image is from a tumor with low *TREM1* expression, the other image illustrates CD15 expression on a tumor with high *TREM1* expression. Scale bars represent 100 μM. **(a)** Symbols show data for n = 8 colorectal cancer patients; lines indicate mean values. Statistical testing was performed with the Wilcoxon matched-pairs signed rank test. *p < 0.05.

We next sought to determine whether TREM-1 is expressed in human colorectal tumors and whether TREM-1 expression in human CRC would also associate with neutrophils. To this end, we performed qRT-PCR on RNA isolated from formalin-fixed paraffin-embedded (FFPE) human CRC tissue specimens and the corresponding non-tumor tissue. In 6/8 cases analyzed, increased mRNA for TREM-1 was indeed detected in the tumor as opposed to the non-tumor tissue (Fig. 5a). The expression pattern of *TREM1* was closely paralleled by the expression of *IL1B* and *S100A9* which were also increased in the tumor tissue (Fig. 5a). In contrast, no differences in mRNA expression levels of *CD14* and *CD68* could be detected between tumor and non-tumor tissues (Fig. 5a). Immunohistochemistry staining for the granulocyte marker CD15 on sections from *TREM1* low- versus *TREM1* high*-*expressing tumors indicated that augmented expression of *TREM1* in human CRC also appeared to associate with increased neutrophil infiltration (Fig. 5b).

## Discussion

TREM-1 is now emerging as a central player not only during acute pathogen-induced reactions but also in chronic and non-infectious inflammatory disorders^20–23^. Pioneering studies have further indicated an involvement of TREM-1 in various types of malignancies, including leukemia^46,47^, NSCLC^27,28,41^, HCC^16,48^ and CRC^29^. Here, using an inflammation-driven model of CRC and mice genetically deficient in *Trem1*, we have established a clear role for TREM-1 in intestinal tumorigenesis, demonstrating that *Trem1* ^−/−^ mice show significantly reduced tumor development.

The attenuated tumor development in *Trem1* ^−/−^ mice may be considered a consequence of their diminished inflammatory response reported in distinct models of experimental colitis^24,25^. However, besides providing direct evidence for a tumor-promoting role of TREM-1 *in vivo*, our study reveals two novel findings: First, in the AOM/DSS model of CRC, tumor-infiltrating neutrophils, but not TAMs, represent the major TREM-1-expressing cell subset. Other reports from distinct tumor models previously described a major role for TREM-1 on TAMs for tumorigenesis^16,27,41^. Second, the absence of TREM-1 affects the intratumoral immune signature with a shift towards an overrepresentation of transcripts related to B cells and Tfh cells in *Trem1* ^−/−^ tumors. In contrast, *Trem1* ^+/+^ tumors exhibited an innate immune signature typically linked to intestinal tumorigenesis, including enhanced expression of pro-inflammatory cytokines and neutrophil-associated markers.

Former studies investigating the potential role of TREM-1 in cancer have either correlated the *in situ* expression of TREM-1 with clinicopathological data^27,48–50^, employed *in vitro* co-cultures of cancer cells with macrophages^27,41^, or have interfered with TREM-1-mediated signaling using *in vivo* tumor models^16,28,29^. Collectively, these findings suggest that distinct tumors can induce the expression of TREM-1 on monocytes/macrophages^27,41,45^ and that expression of TREM-1 in human cancer tissue can serve as a powerful prognostic indicator^27,48–50^. In murine models, the tumor-promoting function of TREM-1 was associated with augmented expression of several pro-inflammatory mediators and increased compensatory proliferation of parenchymal cells^16,29^. In their seminal paper, Wu *et al*. were further able to directly demonstrate that TREM-1-activated Kupffer cells were required and sufficient to promote hepatocellular carcinoma development in response to liver injury^16^.

In view of the proposed significance of TREM-1 expression by macrophages in distinct types of cancer^16,27,41^, it is intriguing that in murine colorectal tumors Ly6C^−^ MHCII^+^ TAMs were evidently TREM-1 negative. This appeared to be a consistent trait and did not reflect a premature time-point of analysis since in more advanced and larger colorectal tumors TAMs equally lacked TREM-1 expression (data not shown). Although cleavage of TREM-1 from activated cells could account for reduced cell surface expression, we did not consider such a scenario a likely explanation for the complete absence of surface TREM-1 on colorectal TAMs, in particular, since TREM-1 was still highly expressed by infiltrating neutrophils and a small subset of Ly6C^+^ MHCII^+^ intermediate monocytes/macrophages. Rather we believe that analogous to the situation in the inflamed colonic mucosa where resident Ly6C^−^ MHCII^+^ macrophages largely retain their hyporesponsive signature and remain TREM-1^low/−^ ^43,44^, macrophages in colorectal tumors are also relatively inert to pro-inflammatory stimuli in their local environment, at least with respect to an upregulation of TREM-1. Alternatively, newly recruited monocytes may downregulate TREM-1 expression because of the TGFβ and IL-10-rich environment of the colonic mucosa or as part of a default tissue-specific differentiation program^42,51^. Unlike the previously described upregulation of TREM-1 on peripheral blood Gr1^+^ monocytes in RMA-S tumor-bearing mice^45^, we did not detect substantial changes in surface TREM-1 expression on any peripheral blood myeloid cell subset isolated from mice with colorectal tumors as opposed to age-matched untreated controls. Similarly, TREM-1 expression on neutrophils was comparable in neutrophils isolated from healthy colonic tissue of untreated control mice, AOM/DSS-induced tumors, as well as adjacent tumor-free, colonic tissues of treated mice (data not shown). Interestingly, a recent study has even reported progressive loss of TREM-1 on tumor-infiltrating monocytes/macrophages in a mouse lung carcinoma model and proposed TREM-1^low^ expression as a novel characteristic for TAMs in human NSCLC tissue^52^. Thus, the expression and functional significance of TREM-1 on cells of the monocyte/macrophage lineage seems to depend on the tissue-dependent context or the nature of tumor-associated inflammation and calls for further investigation across distinct types of cancer.

Although TREM-1 is constitutively expressed by neutrophils and neutrophil-lymphocyte ratios are increasingly incorporated in prognostic scores^53^, to the best of our knowledge a function for TREM-1-expressing neutrophils has so far not been considered in any of the studies addressing the role of TREM-1 in cancer. This may relate to the distinct nature of tumor-eliciting or tumor-associated inflammation in the other types of cancer analyzed so far. In line with the microbial-driven neutrophilic inflammation that is induced by DSS treatment, we demonstrate here that in murine colorectal tumors TREM-1 is mainly expressed by neutrophils, which also represent the predominant myeloid cell population in *Trem1* ^+/+^ tumors. While we could not absolutely quantitate differences in tumor infiltrates between the *Trem1* ^+/+^ or *Trem1* ^−/−^ group (in *Trem1* ^+/+^ mice, tumors had to be pooled as they were too numerous for separate analyses, in *Trem1* ^−/−^ mice, tumors were so small and scarce that they were pooled into a single sample in order to acquire sufficient cells), an increased abundance of neutrophils in *Trem1* ^+/+^ as opposed to *Trem1* ^−/−^ tumors was also strongly suggested by NanoString- and qRT-PCR-based gene expression profiling of individual tumors. Accordingly, irrespective of their size, *Trem1* ^+/+^ tumors exhibited an increased abundance of neutrophil-associated transcripts such as *Itgam*, *Cxcr1*, *Cxcr2* and *S100a8*/*S100a9*. Neutrophil chemotaxis was also identified as a major differentially regulated pathway, since increased levels of *Cxcl1* and *Cxcl2* transcripts were detected in the *Trem1* ^+/+^ tumors. Although the upregulated expression of neutrophil-specific chemokines may only be an indirect consequence of TREM-1 signaling, these data thus provide circumstantial evidence for a TREM-1-dependent recruitment of neutrophils into colorectal tumors in the AOM/DSS model. Indeed, numerous studies involving both spontaneous and inflammation-driven models of CRC have documented the prominent infiltration of murine colorectal tumors with neutrophils and have directly demonstrated beneficial effects of genetic deficiency in *Cxcr2* or targeted neutrophil depletion on tumor development and progression^13,37–39^. The tumor-promoting effects of neutrophils were ascribed to production of nitric oxide radicals contributing to DNA damage^54^, to expression of metalloproteinase (MMP)-9 and neutrophil elastase (NE) driving angiogenesis and enhanced tumor cell proliferation^38^, to myeloid-derived suppressor cell (MDSC) activity and inhibition of CD8^+^ T cell functions^36^, and also to increased pro-inflammatory chemokine and cytokine secretion. Along with augmented expression of neutrophil-associated chemokines and markers, we observed significantly higher levels of transcripts for pro-tumorigenic *Il1b* and *Il6* ^55^ and potential downstream molecules such as *Il17a*, *Il22*, *Jak3* and *Stat3* in *Trem1* ^+/+^ as opposed to *Trem1* ^−/−^ tumors. In addition, in human CRC samples upregulated expression of *TREM1* was associated with increased *IL1B* expression. The pro-tumorigenic effects of IL-1β were previously ascribed to induction of IL-6 secretion by mononuclear phagocytes^13,55^ which was also expressed at higher levels in *Trem1* ^+/+^ tumors (Fig. 2). While we could not directly determine the cellular sources of these cytokines in our model, it is likely that TREM-1-activated neutrophils at least partly contribute to CRC development *via* their augmented secretion of pro-inflammatory factors. The increased induction of chemotactic mediators along with the reported improved survival of neutrophils upon TREM-1 stimulation^25^ likely account for the accumulation of neutrophils in the colons of AOM/DSS-treated *Trem1* ^+/+^ mice. Since presence of TREM-1 mostly seems to impact on the incidence of small tumors rather than the growth of large tumors, this implies that TREM-1-expressing neutrophils may primarily contribute to early tumorigenesis, particularly, as TREM-1 signaling strongly induces iNOS and ROS production^25,56^. However, we believe that the effects of TREM-1-activated neutrophils on tumor development are likely multilayered and operative also in established tumors.

One limitation of the AOM/DSS model of CRC is that tumors do not generally metastasize in C57BL/6 mice^3^. Therefore, we were not able to address the previously postulated association between TREM-1 and cancer progression^27,49,50^ with our *in vivo* model. However, we found that the immune signature of *Trem1* ^+/+^ and *Trem1* ^−/−^ tumors not only differed with respect to the increased expression of pro-inflammatory genes in *Trem1* ^+/+^ tumors, but that *Trem1* ^−/−^ tumors were characterized by an increased abundance of transcripts related to adaptive immune stimulation, B cells and Tfh cells. The expression of these markers and transcription factors such as *Ikzf1*, *Pax5* and *Rorc* is reminiscent of tertiary lymphoid tissue (TLT) formation which has been associated with a better prognosis in early-stage CRC^57^. Indeed, in their comprehensive study on the human intratumoral immune landscape, Bindea *et al*., established CXCR5/CXCL13 and the Tfh/B cell axis as major antitumor players and powerful markers for predicting prolonged patient survival^6^. It is currently unclear why the *Trem1* ^−/−^ tumor environment favors an accumulation of B cells. This phenomenon may relate to the decreased genomic stability of *Cxcl13* ^6^ or to a retinoic acid deficit^58^ in the context of the increased intestinal inflammation in *Trem1* ^+/+^ tumors as both factors have been implicated in lymphoid tissue formation^59^. However, further investigation will be necessary to conclusively address this aspect.

Notably, RNA-sequencing analysis of human CRC specimens at various stages has identified *TREM1* as one of two predominant regulators activated during CRC tumorigenesis and a risk score based on nineteen genes regulated by *TREM1* and *CTGF* represented a significant prognostic indicator for CRC aggressiveness^50^. Hence, the expression and prognostic significance of TREM-1 in human CRC clearly warrants further investigation.

In summary, our study demonstrates that TREM-1 critically contributes to inflammation-driven intestinal tumorigenesis. Our data may further challenge the current notion that TREM-1^+^ macrophages may be the critical players across all types of cancer by identifying neutrophils as the major TREM-1-expressing cell subset in the AOM/DSS model of inflammation-induced CRC. While the precise mechanisms behind TREM-1-mediated tumor development still need to be deciphered, our findings show that intratumoral expression of *Trem1* associates with increased expression of pro-inflammatory genes implicated in intestinal tumorigenesis. Considering the well-established link between innate inflammation and cancer, TREM-1 may emerge as a powerful prognostic indicator not only in CRC but across several types of cancer. Finally, our results suggest that therapeutic inhibition of TREM-1 may not only prevent onset of cancer in patients with IBD, but that TREM-1 could represent a novel immunotherapeutic target to restrain cancer-promoting inflammation without jeopardizing anti-microbial and anti-tumor immunity.

## Materials and Methods

### Mice

*Trem1* ^−/−^ mice (on the C57BL/6 background) were generated in our laboratory as described previously^25^. *Trem1* ^+/+^ wildtype mice represented former littermates of *Trem1* ^−/−^ mice. *Trem1* ^+/+^ and *Trem1* ^−/−^ mice were bred and maintained in isolated ventilated cages at the central animal facility of the University of Bern. Experiments were initiated with 10 weeks old sex-matched *Trem1* ^+/+^ and *Trem1* ^−/−^ mice. Prior to and during the experiments, soiled bedding was exchanged to match commensal communities between experimental groups. Experiments were performed in accordance with Swiss Federal regulations and were approved by the Cantonal Veterinary Office.

### Induction and assessment of AOM/DSS-induced colorectal cancer

As a model for murine CRC, we used the AOM/DSS model which included in our protocol two i.p. injections of AOM (Sigma, Switzerland, Cat. No. A5486) and three cycles of 1% DSS (MP Biomedicals, Cat. No. 160110) administered in the drinking water^60^. Briefly, mice were injected with 10 mg/kg AOM and rested for 6–7 days prior to the 1^st^ DSS cycle (5 days). After 6–7 days on regular water, mice received a 2^nd^ injection of AOM and an additional 3-day rest on water before the 2^nd^ DSS cycle (4 days). Thereafter, mice were rested for up to 13 days before the 3^rd^ DSS cycle (3 days). From this, mice were placed on water until approximately 80 days post the initial AOM injection. Throughout the entire course of the experiment, mice were monitored regularly for their overall well-being and changes in their body weight. Macroscopic tumors were counted independently by two observers. Tumor load was measured with a pair of sliding calipers and calculated as (W × L × H) × π/6 as previously described^60^. Formalin-fixed paraffin-embedded and H&E stained tumors sections were graded by a board-certified pathologist in a blinded manner according to criteria defined in the legend to Supplementary Figure 1.

### RNA isolation and gene expression analysis

From each mouse, one randomly selected tumor was placed in RNAlater (Life Technologies, USA). For RNA isolation, tumors were transferred into TRI Reagent (Sigma) and tissues were homogenized with a TissueLyser (Qiagen). RNA was isolated according to the manufacturer’s instructions. For NanoString-based gene expression profiling, the nCounter Mouse Immunology panel was used (NanoString technologies, Seattle, USA). Samples were analyzed at the Genomics Platform of the University of Geneva (GE3, University of Geneva, Switzerland). mRNA expression of particular genes of interest was confirmed by qRT-PCR. Briefly, contaminating genomic DNA was digested with DNase I (Ambion) and cDNA was generated with the High Capacity Reverse Transcription Kit (Applied Biosystems). qRT-PCR reactions were set up with a SYBR-green based reaction mix (Roche, Switzerland) and Quantitect primers (Qiagen) and analyzed on a 7500 Real-time PCR System (Applied Biosystems). Expression of candidate genes was normalized to the house-keeping gene *Gapdh*, multiplied by a factor of 1000 and depicted as relative amount of mRNA (to *Gapdh*).

### Cell isolation from murine colons and tumors

Tumor-free colons and colon sections were opened longitudinally and cut into small pieces. Epithelial cells were removed by incubation of tissue pieces in HBSS/HEPES containing 5% FCS, 2 mM EDTA for 2 × 15 min at 37 °C under magnetic stirring. After washing of tissue pieces with EDTA-free buffer, lamina propria (LP) cells were obtained by digestion with 100 U/ml Collagenase (Type IV, Sigma) and 50 U/ml DNase (Type I, grade II, Roche) in Ca^2+^ and Mg^2+^-supplemented HBSS/HEPES 5% FCS for 20–30 min at 37 °C. For isolation of cells from tumors, tumors were carefully excised from the tumor-free mucosa and transferred into 15 ml U-bottom tubes with small magnetic stirrers (larger tumors were additionally minced with a scalpel). Pooled tumors were subsequently digested in Ca^2+^ and Mg^2+^-supplemented HBSS/HEPES 5% FCS with 100 U/ml collagenase (Type IV), 50 U/ml DNase (Type I) and 150 U/ml hyaluronidase (Type V, Sigma) for 45′ at 37 °C. Cell suspensions from tumors and tumor-free colons were passed through a 70 μM cell strainer and further characterized by flow cytometry.

### Antibodies and flow cytometry

Cell suspensions were Fc-Receptor blocked and stained with the following antibodies (all from Biolegend unless indicated otherwise): (Myeloid panel) anti-Ly6G-FITC (1A8; 1.25 μg/ml), anti-CD64-PE (X54-5/7.1; 2 μg/ml), anti-CD11c-PerCP-Cy5.5 (N418; 1 μg/ml), anti-CD11b-PE-Cy7 (M1/70; 0.2 μg/ml), anti-Ly6C-AlexaFluor700 (HK1.4; 2 μg/ml), anti-MHCII-APC-Cy7 (M5/114.15.2; 0.067 μg/ml), anti-TREM-1-eFluor660 (TR3MBL1 from eBioscience; 0.2 μg/ml), anti-CD45-BrilliantViolett (30-F11; 1 μg/ml). (Lymphocyte panel) anti-CD8b-FITC (53-5.8; 0.8 μg/ml), anti-TCRab-PE, anti-GL7-PerCP-Cy5.5 (GL7; 1 μg/ml), anti-CD19-PE-Cy7 (6D5; 0.5 μg/ml), anti-CD11b-PacificBlue (M1/70; 0.25 μg/ml), anti-NK1.1-AlexaFluor700 (PK136; 2.5 μg/ml), anti-CD4-APC-Cy7 (RM4-5; 0.25 μg/ml), anti-BTLA-AlexaFluor647 (6A6; 2.5 μg/ml), anti-CD45-BrilliantViolett (30-F11; 1 μg/ml). As an isotype control for the anti-TREM-1-e-Fluor660 a mouse anti rat IgG2a-eFluor660 antibody (eBR2a; 0.2 μg/ml) from eBioscience was used. Dead cells were excluded using DAPI (Invitrogen) at a final concentration of 0.5 μg/ml. All samples were acquired on a LSRII SORP (BD Biosciences, San Diego, CA) and analyzed using the FlowJo Software (Tree Star, Ashland, OR).

### Human CRC tissue specimens

Human FFPE CRC tissue specimens were collected at the Institute of Pathology, University of Bern and analyzed in concordance with Swiss regulations for retrospective research on human tissue specimens collected for diagnostic purposes before 06/2014 and following the approval by the Ethics Committee Bern for which written consent was not required prior to the implementation of the Swiss law on human research (“Humanforschungsgesetz”) in 2014.

Suitable CRC specimens and corresponding tumor-free normal mucosa were selected from a total of eight patients based on the prior analysis of the case history and H&E-stained sections. RNA from five 10 μM thick FFPE tissue sections was purified using a column-based approach described by Oberli *et al*.^61^. SYBR-green based qRT-PCR was performed with QuantiTect (Qiagen) primers as described above. Immunostainings for CD15 were performed on an automated platform (Leica Bond RX, Leica Biosystems, Switzerland) using the anti-CD15 monoclonal antibody clone MMA (BD Biosciences).

### Statistical analysis

All data except for NanoString data were analyzed using Prism software version 6.0c. Statistical tests were performed as described in the figure legends. In general, the D’Agostino & Pearson omnibus K2 normality test was performed to determine whether the samples were distributed according the Gaussian distribution. For normally-distributed samples the unpaired t test was performed; for samples without Gaussian distribution, the Mann Whitney t test was used. Only statistically significant differences are indicated in the figures. For analysis of the NanoString data, significant differences in gene expression were calculated between *Trem1* ^+/+^ and *Trem1* ^−/−^ tumors. Gene expression values were first log-transformed. The Bioconductor limma package was used to perform a moderate t-test on all genes^62^. Pathway analysis was performed using the SetRank tool^63^. The heat-map was constructed in R.

### Availability of data

All relevant data that support the findings of this study, including the full NanoString gene expression profiling data, are available from the corresponding authors upon request.

## Electronic supplementary material

Supplementary Information
