# Supplementary material for: TREM-1 promotes intestinal tumorigenesis
Source: Sci Rep. 2017 Nov 1;7:14870. doi: 10.1038/s41598-017-14516-4 (PMC5665947; doi:10.1038/s41598-017-14516-4)
Supplement: Supplementary file 1 — Supplementary Information [file 41598_2017_14516_MOESM1_ESM.pdf]

Supplementary Information

TREM-1 promotes intestinal tumorigenesis

Leslie Saurer[1+], Daniel Zysset[1+], Silvia Rihs[1], Lukas Mager[1,2], Matteo Gusberti[1], Cedric Simillion[3,4], Alessandro Lugli[1], Inti Zlobec[1], Philippe Krebs[1*], Christoph Mueller[1*]

[1]Institute of Pathology, University of Bern, Switzerland

[2]Department of Physiology and Pharmacology, Cumming School of Medicine, University of Calgary, Canada

[3]Department of Clinical Research, University of Bern, Switzerland

[4]Interfaculty Bioinformatics Unit and SIB Swiss Institute of Bioinformatics, University of Bern, Switzerland

Supplementary Figure 1

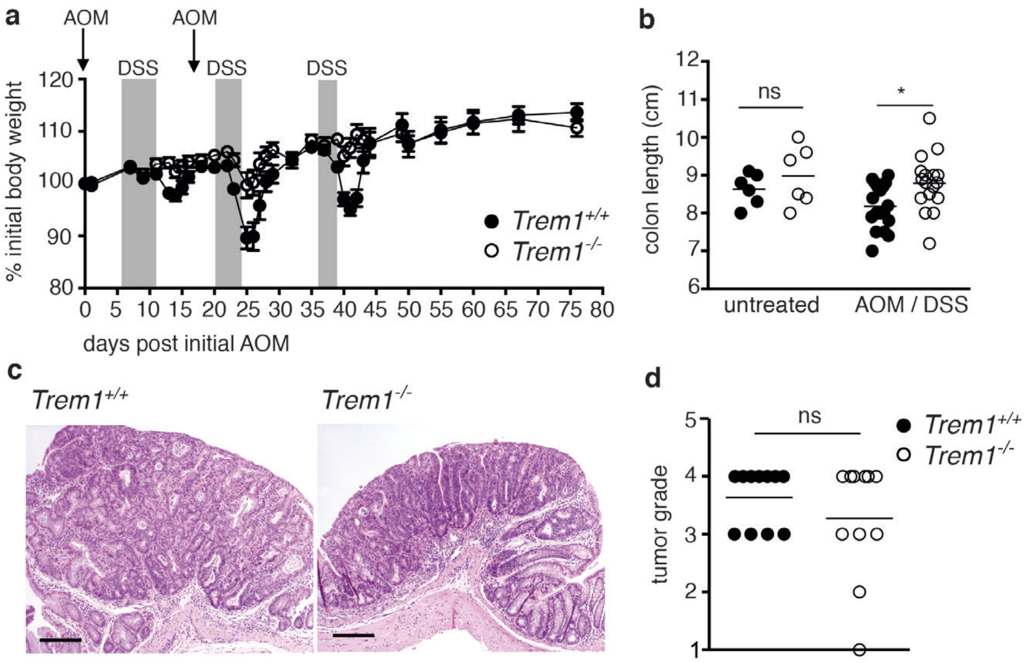

Supplementary Figure 1

Clinical and histopathological data of AOM/DSS-treated *Trem1*$^{+/+}$ and *Trem1*$^{-/-}$ mice.

(a) Relative body weight (expressed as % of initial body weight) of *Trem1*$^{+/+}$ and *Trem1*$^{-/-}$ mice during the AOM/DSS treatment protocol. Symbols show mean values of n=11-12 mice per group up to the 3rd DSS cycle and n=6-7 mice per group for the remaining time-period. Error bars indicate the SEM. Data from one of two independent experiments are shown. **(b)** Colon length at 80 days post initial AOM injection in AOM/DSS-treated mice and age-matched untreated control mice. **(c)** Images of H&E-stained sections of a representative tumor isolated from a *Trem1*$^{+/+}$ versus *Trem1*$^{-/-}$ mouse (both tumors: grade 4). Scale bars represent 200 μM. **(d)** Histopathological assessment of tumor grade in *Trem1*$^{+/+}$ versus *Trem1*$^{-/-}$ mice. Symbols indicate the highest tumor grade per colon. Grade 1: Adenoma with mild dysplasia (low grade); Grade 2: Adenoma with moderate dysplasia (low grade); Grade 3: Adenoma with severe dysplasia (high grade); Grade 4: Adenoma with high-grade dysplasia and infiltration of the lamina propria (intramucosal neoplasia); (Grade 5: invasive carcinoma). Statistical testing was performed with 2way ANOVA **(b)** and the Mann-Whitney test **(d)**.

Supplementary Figure 2

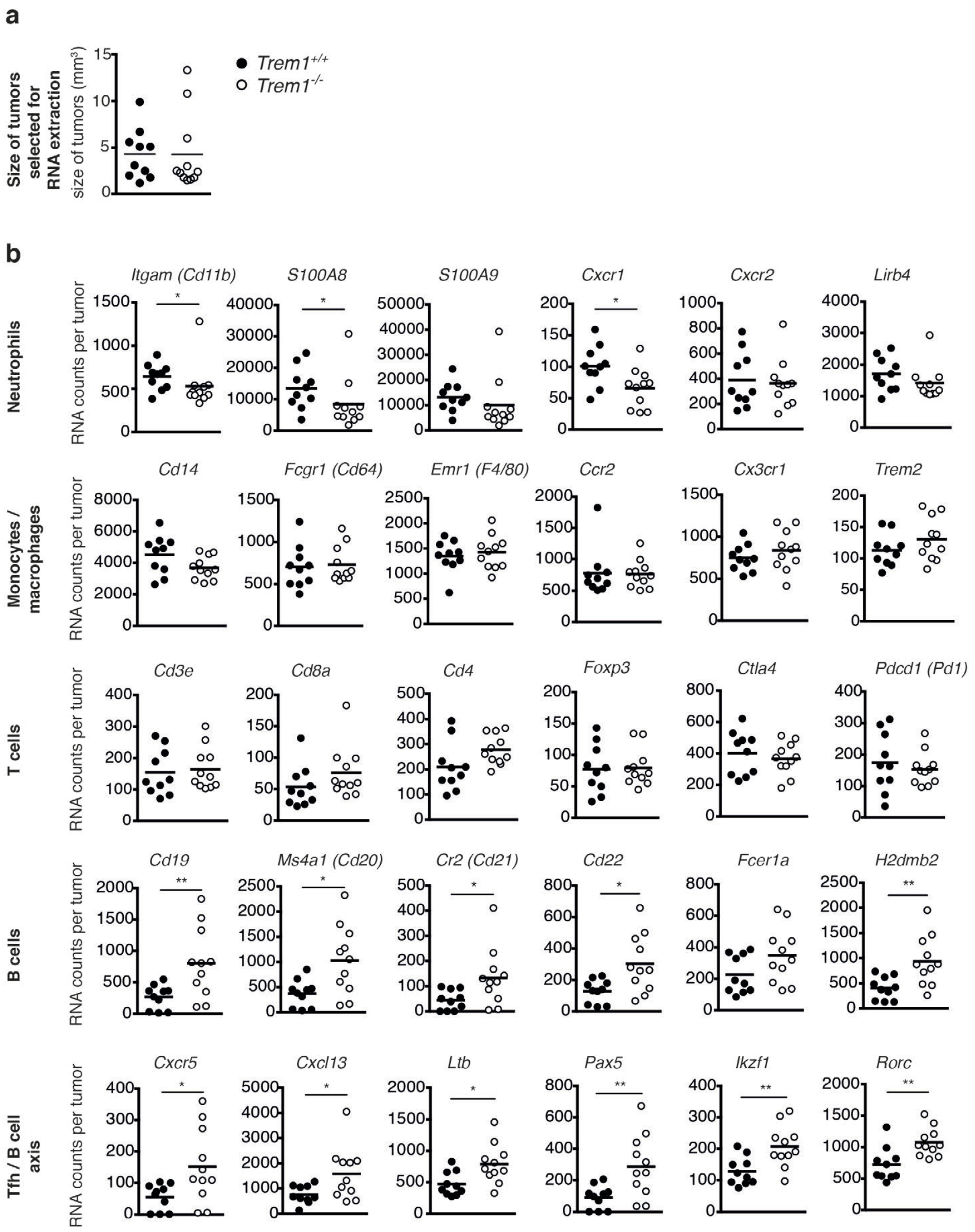

Supplementary Figure 2

Size distribution of tumors used in gene expression profiling and expression of genes associated with particular immune cell subsets.

(a) Size distribution of tumors that were selected for gene expression profiling. One tumor was selected per mouse **(b)** Expression of genes associated with particular immune cell subsets (neutrophils, monocytes/macrophages, T cells, B cells and the Tfh/B cell axis) as determined by NanoString-based analysis. Symbols show data for individual mice, lines indicate mean values per group. Statistical testing was performed with the unpaired t-test or Mann-Whitney test. *, $p < 0.05$; **, $p < 0.01$.

Supplementary Figure 3

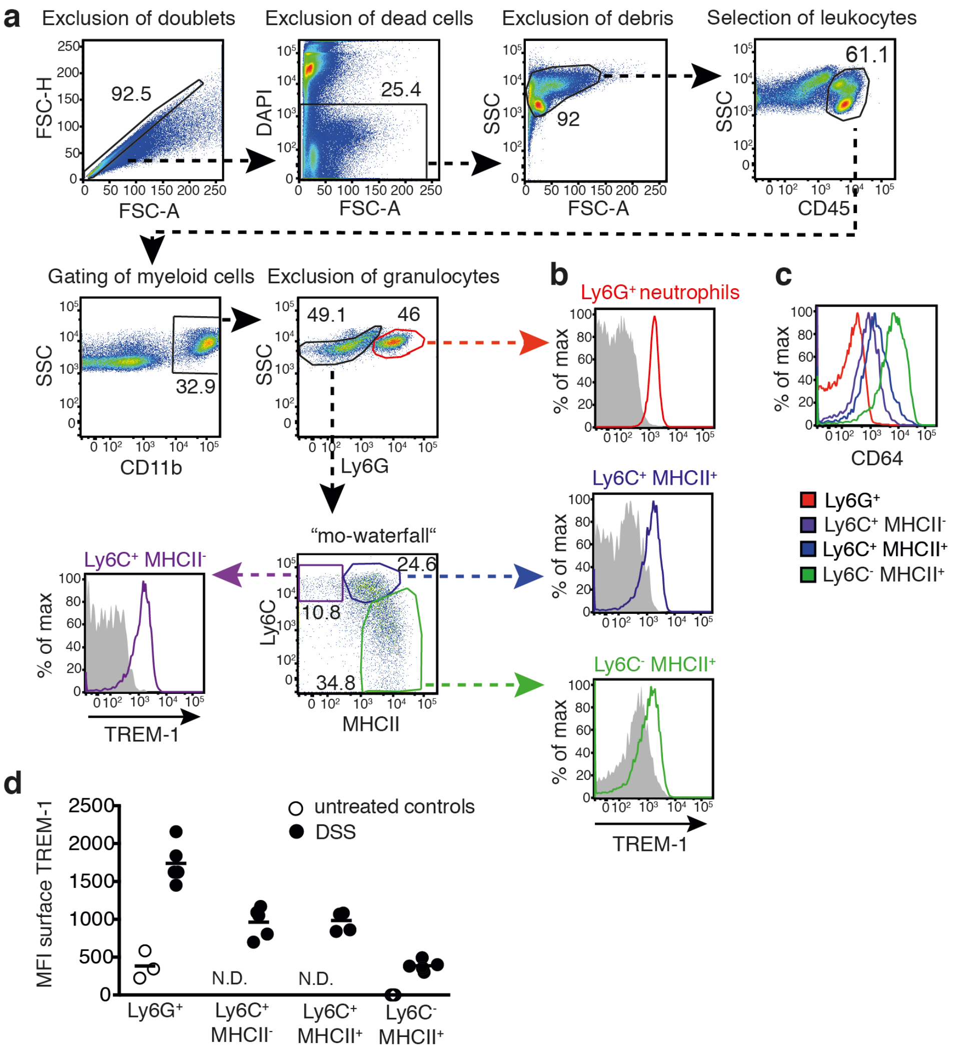

Supplementary Figure 3

Gating strategy for identification of colonic myeloid cell subsets.

(a) Initial gating included exclusion of doublets, dead cells and debris followed by gating of CD45$^+$ leukocytes and subgating of CD11b$^+$ cells. Among CD11b$^+$ cells, granulocytes were excluded by gating of Ly6G$^-$ as opposed to Ly6G$^+$ cells; gating of Ly6G$^-$ cells also excluded SSC^{high} eosinophils. Among Ly6G$^-$ CD11b$^+$ cells, three distinct populations were identified according to the "monocyte waterfall": Ly6C$^+$ MHCII$^-$ monocytes, Ly6C$^+$ MHCII$^+$ intermediate monocytes/macrophages and Ly6C$^-$ MHCII$^+$ macrophages. **(b)** Expression of surface TREM-1 (lines; filled histograms show isotype control-stained cells) by the indicated subsets. **(c)** Expression of CD64 by the indicated subsets. Ly6C$^-$ MHCII$^+$ cells were CD64$^+$ and did not include CD64$^-$ dendritic cells. **(d)** Mean fluorescence intensity (MFI) of TREM-1 surface expression (with values for isotype control-stained cells subtracted) on the indicated cell subsets in untreated control *Trem1*$^{+/+}$ mice (n=3) or in *Trem1*$^{+/+}$ mice analyzed after the 3rd DSS cycle (n=5). N.D., cell subset not detected.

Supplementary Figure 4

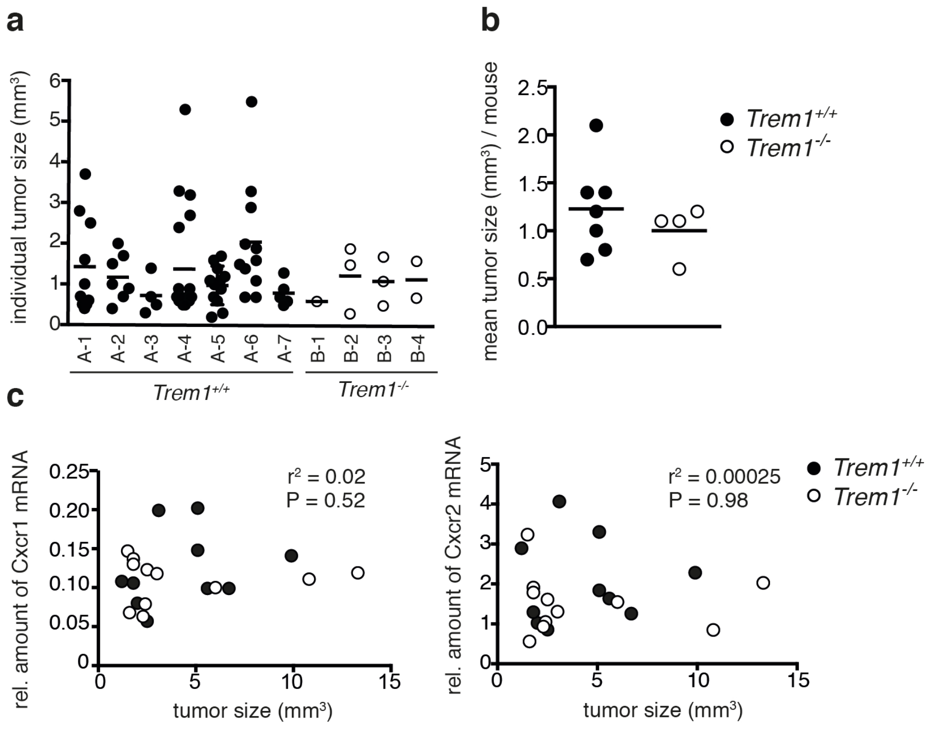

Supplementary Figure 4

Size distribution of tumors selected for flow cytometry analysis and correlation of tumor size with expression of neutrophil markers.

a) Size of individual tumors which were collected for FACS analysis. Black symbols represent tumors from *Trem1*$^{+/+}$ mice (n=7) and white symbols represent tumors from *Trem1*$^{-/-}$ mice (n=4). **b)** Mean tumor size (per mouse) of tumors collected for flow cytometry. In the *Trem1*$^{+/+}$ group, tumors per colon were pooled to generate one tumor sample per mouse. In the *Trem1*$^{-/-}$ group, all tumors were pooled to generate a single sample. **c)** No significant correlation was found for the size of individual tumors and mRNA expression of the neutrophil markers *Cxcr1* and *Cxcr2*.

Supplementary Table 1

List of genes that were differentially expressed (adj. P-Value < 0.1) between *Trem1*$^{+/+}$ and *Trem1*$^{-/-}$ tumors

Symbol	gene name	log FC	adj. P-value
Trem1	triggering receptor expressed on myeloid cells 1	-7.96	9.56E-12
Ccl7	chemokine (C-C motif) ligand 7	-1.40	0.00096
Ccl2	chemokine (C-C motif) ligand 2	-1.08	0.01756
Il1b	interleukin 1 beta	-0.81	0.01756
Jak3	Janus kinase 3	-0.55	0.01756
Il22	interleukin 22	-3.20	0.01950
Socs3	suppressor of cytokine signaling 3	-0.83	0.02843
Mapk11	mitogen-activated protein kinase 11	-0.83	0.03044
Il3	interleukin 3	-1.67	0.03210
Cd3eap	CD3E antigen, epsilon polypeptide associated protein	-0.63	0.03346
Ccl4	chemokine (C-C motif) ligand 4	-1.07	0.03492
Cxcr1	chemokine (C-X-C motif) receptor 1	-1.09	0.04231
Il18rap	interleukin 18 receptor accessory protein	-0.75	0.04607
Fn1	fibronectin 1	-0.45	0.04781
Nfkbia	nuclear factor of kappa light polypeptide gene enhancer in B cells inhibitor, alpha	-0.31	0.04781
Il17f	interleukin 17F	-1.85	0.04810
Cd46	CD46 antigen, complement regulatory protein	-1.72	0.04810
Nod2	nucleotide-binding oligomerization domain containing 2	-0.55	0.05398
Il6	interleukin 6	-2.59	0.05443
Itgam	integrin alpha M	-0.46	0.05443
Lif	leukemia inhibitory factor	-0.67	0.05968
Il17a	interleukin 17A	-2.21	0.06379
Clec4e	C-type lectin domain family 4, member e	-0.78	0.07586
Pdgfb	platelet derived growth factor, B polypeptide	-0.45	0.07586
Cebpb	CCAAT/enhancer binding protein (C/EBP), beta	-0.34	0.07921
Il13	interleukin 13	-1.18	0.08132
Tgfbi	transforming growth factor, beta induced	-0.26	0.08792
Stat3	signal transducer and activator of transcription 3	-0.20	0.08792
Sele	selectin, endothelial cell	-1.52	0.09183
Tnfsf11	tumor necrosis factor (ligand) superfamily, member 11	-0.70	0.09183
Traf5	TNF receptor-associated factor 5	0.41	0.01756
Vcam1	vascular cell adhesion molecule 1	0.48	0.01756
Ikzf1	IKAROS family zinc finger 1	0.55	0.01756
Btla	B and T lymphocyte associated	1.11	0.01756
Cd55	CD55 antigen	0.42	0.01950
Ncf4	neutrophil cytosolic factor 4	0.31	0.02843
Icosl	icos ligand	0.40	0.02843
Cd160	CD160 antigen	1.76	0.02843
Cd2	CD2 antigen	0.57	0.03044
NA	NA	0.38	0.03571
Mx1	MX dynamin-like GTPase 1	0.47	0.03571
H2-DMb2	histocompatibility 2, class II, locus Mb2	1.26	0.03689
Cd97	CD97 antigen	0.37	0.04810
Ifna1	interferon alpha 1	2.78	0.04810
Ltb	lymphotoxin B	0.66	0.05443
Cd53	CD53 antigen	0.40	0.07113
Ccr6	chemokine (C-C motif) receptor 6	1.17	0.08132
Itga4	integrin alpha 4	0.30	0.08446
Pecam1	platelet/endothelial cell adhesion molecule 1	0.25	0.08988
Arhgdib	Rho, GDP dissociation inhibitor (GDI) beta	0.40	0.08988

Supplementary Table 2

Pathways that were differentially regulated between *Trem1*$^{+/+}$ and *Trem1*$^{-/-}$ tumors

Name	description	database	size	adj. P-Value
GO:0030593	neutrophil chemotaxis	GOBP	19	0.0056
ko04668	TNF signaling pathway	KEGG	44	0.0056
GO:0045123	cellular extravasation	GOBP	12	0.0056
GO:0061032	visceral serous percardium development	GOBP	2	0.0056
GO:0035872	nucleotide-binding domain, leucine rich repeat	GOBP	3	0.0056
GO:0031727	CCR2 chemokine receptor binding	GOMF	3	0.0070
ko04664	Fc epsilon RI signaling pathway	KEGG	16	0.0272
GO:0031225	anchored component membrane	GOCC	17	0.0311
